# Building a Safe and Transparent Workflow for Large Language Model (LLM)-Assisted Clinical Trials and Prediction Models: A Technical Report

**DOI:** 10.7759/cureus.92571

**Published:** 2025-09-17

**Authors:** João Frutuoso

**Affiliations:** 1 Critical Care, Unidade Local de Saúde Lisboa Ocidental, Lisbon, PRT; 2 Management, Natura Clinica Medica, Lisbon, PRT

**Keywords:** generative ai, large language models, publication ethics, reporting guidelines, research integrity

## Abstract

The use of large language models (LLMs) in clinical trials and prediction models is expanding rapidly, offering opportunities for efficiency but also raising concerns about privacy, fairness, accuracy, and accountability. This technical report proposes a structured workflow to support research teams in adopting LLMs while preserving scientific standards and public trust. The workflow is organized into seven sequential steps: (i) scope definition and governance, (ii) retrieval-augmented literature review, (iii) model evaluation and benchmarking, (iv) documentation and audit trail, (v) expert quality gates, (vi) manuscript disclosure, and (vii) privacy and security safeguards.

To facilitate adoption, we provide reusable checklists that map study types to relevant international reporting guidelines, including Consolidated Standards of Reporting Trials - Artificial Intelligence (CONSORT-AI), Standard Protocol Items: Recommendations for Interventional Trials - Artificial Intelligence (SPIRIT-AI), Transparent Reporting of a Multivariable Prediction Model for Individual Prognosis or Diagnosis - Artificial Intelligence (TRIPOD+AI), Preferred Reporting Items for Systematic Reviews and Meta-Analyses (PRISMA), and Developmental and Exploratory Clinical Investigations of Decision - Support Systems Driven by Artificial Intelligence (DECIDE-AI). The framework is designed to mitigate risks such as fabricated citations, biased outputs from skewed datasets, and over-reliance on automated text. Rather than replacing human reasoning, it aims to augment it, offering greater speed while maintaining accountability, reproducibility, and transparency.

By combining governance rules, technical safeguards, and human oversight, this workflow provides a practical and auditable path for integrating LLMs into clinical trials and prediction models without eroding confidence in scientific work.

## Introduction

Large language models (LLMs) are rapidly entering medical research workflows and, in health contexts, require careful governance to safeguard rigor and public trust [1-3]. Global normative frameworks from the United Nations Educational, Scientific and Cultural Organisation (UNESCO) and the Organisation for Economic Co-operation and Development (OECD) outline principles of transparency, accountability, and risk management that are directly applicable to research settings [2,3].

Editorial policies have converged on shared expectations. The International Committee of Medical Journal Editors (ICMJE) requires transparent disclosure of artificial intelligence (AI) assistance and reaffirms that humans remain accountable for the work; the World Association of Medical Editors (WAME) provides operational recommendations for responsible use of generative AI in manuscripts; and the Committee on Publication Ethics (COPE) clarifies that AI systems cannot be credited as authors [4-6]. These positions have practical implications for how research teams plan, document, and report any AI-assisted activity.

Specialized reporting guidance has also emerged across study types. Interventional trials and their protocols are covered by the Consolidated Standards of Reporting Trials - Artificial Intelligence (CONSORT-AI) and the Standard Protocol Items: Recommendations for Interventional Trials - Artificial Intelligence (SPIRIT-AI), which specify intervention details, oversight, and safeguards [7,8]. Early, live clinical evaluation of AI-enabled decision support is addressed by Developmental and Exploratory Clinical Investigations of Decision-Support Systems Driven by Artificial Intelligence (DECIDE-AI), capturing usability, human factors, and safety [9]. For prediction models and systematic reviews, the Transparent Reporting of a Multivariable Prediction Model for Individual Prognosis or Diagnosis - Artificial Intelligence (TRIPOD+AI) and the Preferred Reporting Items for Systematic Reviews and Meta-Analyses 2020 (PRISMA 2020) set expectations for transparent development, validation, and evidence synthesis [10,11]. Recent commentaries in Nature Medicine and other journals have highlighted that while these frameworks are essential, they remain fragmented and insufficient without practical workflows for day-to-day implementation [12].

Despite advances, recurrent risks persist. Models trained on skewed data can amplify inequities, and weak oversight may lead to unintended consequences or over-reliance on automation [13,14]. Editorials have additionally warned that general-purpose chatbots can fabricate content and citations if used without safeguards, undermining transparency and reproducibility [15-17]. To strengthen factual grounding, retrieval-augmented generation (RAG) limits generation to rights-cleared, authoritative sources, and recent medicine-specific benchmarks suggest measurable accuracy gains when retrieval is well designed [18,19].

This technical report translates the above expectations into a practical, auditable workflow for day-to-day research use. We provide a seven-step process covering governance, literature/retrieval, documentation, human quality gates, and privacy; three reusable checklists that map study types to appropriate reporting guidance; and a figure-grade summary to facilitate implementation and audit.

## Technical report

Table 1 summarizes the major frameworks most relevant to LLM-assisted clinical trials and prediction models, including their scope, focus areas, and applicability. Our proposed workflow is designed to integrate and operationalize these guidelines, translating their high-level recommendations into a reproducible, auditable, seven-step process for research teams.

**Table 1:** Comparative summary of the main international reporting frameworks applicable to AI in clinical trials and prediction models. Each framework addresses a distinct study design and together provides complementary expectations for transparency, reproducibility, and safety. The proposed workflow operationalizes these principles into a unified seven-step process. AI: artificial intelligence, LLM: large language model, PRISMA: Preferred Reporting Items for Systematic Reviews and Meta-Analyses, RCT: randomized controlled trial, SPIRIT-AI: Standard Protocol Items: Recommendations for Interventional Trials - Artificial Intelligence, TRIPOD+AI: Transparent Reporting of a Multivariable Prediction Model for Individual Prognosis or Diagnosis - Artificial Intelligence; CONSORT-AI: Consolidated Standards of Reporting Trials - Artificial Intelligence, DECIDE-AI: Developmental and Exploratory Clinical Investigations of Decision - Support Systems Driven by Artificial Intelligence,

Framework	Scope	Key focus areas	Relevance for LLM-assisted research
CONSORT-AI (2020)	RCTs involving AI interventions	Trial design, intervention description, participant flow, handling of AI outputs, reproducibility	Ensures that any RCT using LLMs (e.g., clinical decision support tools) transparently specifies model version, training data context, and oversight mechanisms
SPIRIT-AI (2020)	Protocols for interventional trials with AI	Pre-specification of AI intervention details, governance, human oversight, monitoring of safety	Provides structure for trial protocols involving LLM-based tools, requiring upfront definition of model role, risks, and contingency plans
DECIDE-AI (2021)	Early-phase, live clinical evaluation of AI-enabled systems	Usability, human factors, workflow integration, real-world safety	Relevant when piloting LLMs for bedside tasks (e.g., clinical note drafting, adverse event detection), focusing on clinician interaction and safety monitoring
TRIPOD+AI (2023, in development from TRIPOD+AI)	Prediction model development and validation	Transparent description of data, predictors, outcomes, model performance, validation	Applicable for LLM-based prognostic/predictive models, requiring disclosure of training/testing datasets and performance benchmarking
PRISMA 2020 (with AI considerations)	Systematic reviews and meta-analyses	Search strategies, inclusion/exclusion criteria, reproducibility of evidence synthesis	Relevant when LLMs are used for literature screening or evidence synthesis; mandates clear documentation of how automation was applied

Governance and scope

Define which tasks are appropriate for LLM assistance (e.g., outlining, language refinement, and retrieval-augmented generation (RAG)-based summarization) and which are excluded; record this in a governance note and in Table 2. For interventional trials and their protocols, align reporting with CONSORT-AI and SPIRIT-AI so that intervention details, oversight, and safeguards are pre-specified [7,8]. For early, live clinical evaluation of AI-enabled decision support, use DECIDE-AI to capture usability, human factors, and safety elements [9]. For clinical prediction models and systematic reviews, follow TRIPOD+AI and PRISMA 2020 across development/validation and evidence synthesis, respectively [10,11]. Recent commentaries in Nature Medicine have highlighted that while these frameworks are essential, they remain fragmented and insufficient without practical workflows for day-to-day implementation [12].

**Table 2:** Governance and scope checklist. These checklists are intended to be completed and filed with the study’s protocol and submission package. They support transparency and do not replace formal reporting guidelines (CONSORT‑AI, SPIRIT‑AI, DECIDE‑AI, and TRIPOD+AI). BAA: business associate agreement, DPA: data processing agreement, GDPR: General Data Protection Regulation, LLM: large language model, PII: personally identifiable information, PHI: protected health information, RAG: retrieval-augmented generation, OSF: Open Science Framework; SPIRIT-AI: Standard Protocol Items: Recommendations for Interventional Trials - Artificial Intelligence, TRIPOD+AI: Transparent Reporting of a Multivariable Prediction Model for Individual Prognosis or Diagnosis - Artificial Intelligence; CONSORT-AI: Consolidated Standards of Reporting Trials - Artificial Intelligence, DECIDE-AI: Developmental and Exploratory Clinical Investigations of Decision - Support Systems Driven by Artificial Intelligence

Checklist item	What to document (audit trail)	Pass criteria (Y/N/NA)
Define LLM-assisted tasks	List of allowed tasks (e.g., outlining, editing, RAG summarization) and explicit exclusions	Tasks enumerated; exclusions stated
Data sensitivity screening	Data classification (patient PII/PHI/other sensitive), de-identification status	High-risk data kept off public tools
Legal/ethical basis	Ethics review need (Y/N), legal basis (e.g., GDPR Art. 6/9 if applicable)	Basis recorded; approvals filed
Institutional approval and agreements	DPA/BAA or institutional approval for chosen tool/provider	Agreements in place before use
Model identity and version	Model/provider, version/date, hosting, and data residency	Fully recorded
Access control	Who can access the tool/corpus; authentication method	Named users; role-based access
Prompt hygiene	Policy to prohibit entry of protected/identifiable data into public tools	Policy documented and communicated
Disclosure plan	Where/how AI use will be disclosed (methods/acknowledgments/cover letter)	Location defined
Roles and responsibilities	Human owners for literature, data extraction, statistics, references	Named individuals assigned
Bias/fairness plan	Pre-specified subgroups and fairness checks	Subgroups defined
Security posture	Statement of security controls (encryption at rest/in transit, audit logging)	Controls described
Source restrictions	Approved sources/corpora for RAG (licensed, open, institutional)	Approved sources list/allow-list defined
Pre-registration (if applicable)	Registry/OSF/PROSPERO/SPIRIT-AI details	Identifier recorded
Exit/opt-out (if applicable)	Process to remove specific data from pipelines	Documented

Beyond reporting standards, anticipate fairness risks: skewed training data can exacerbate inequities, and weak oversight may foster over-reliance or fabricated content, undermining transparency and reproducibility [13-16]. In addition, some authors rely on “black box” deep research approaches, where neither the provenance of outputs nor the model’s internal logic is accessible. Such practices heighten concerns over reproducibility and accountability, underscoring the importance of explicit governance and transparent audit trails.

To strengthen factual grounding, pair generation with retrieval-augmented workflows restricted to rights-cleared, authoritative sources; recent medicine-specific evaluations, such as the MIRAGE benchmark (Benchmarking Retrieval-Augmented Generation for Medicine), suggest accuracy gains when retrieval is well designed [18,19].

Where health data are involved, enforce strict privacy boundaries: keep protected/identifiable information off public tools, prefer institution-approved solutions under appropriate agreements, and document the legal basis, as in the General Data Protection Regulation (GDPR) and any required approvals (see Table 2) [1,20].

Practical workflow

**Figure 1:**
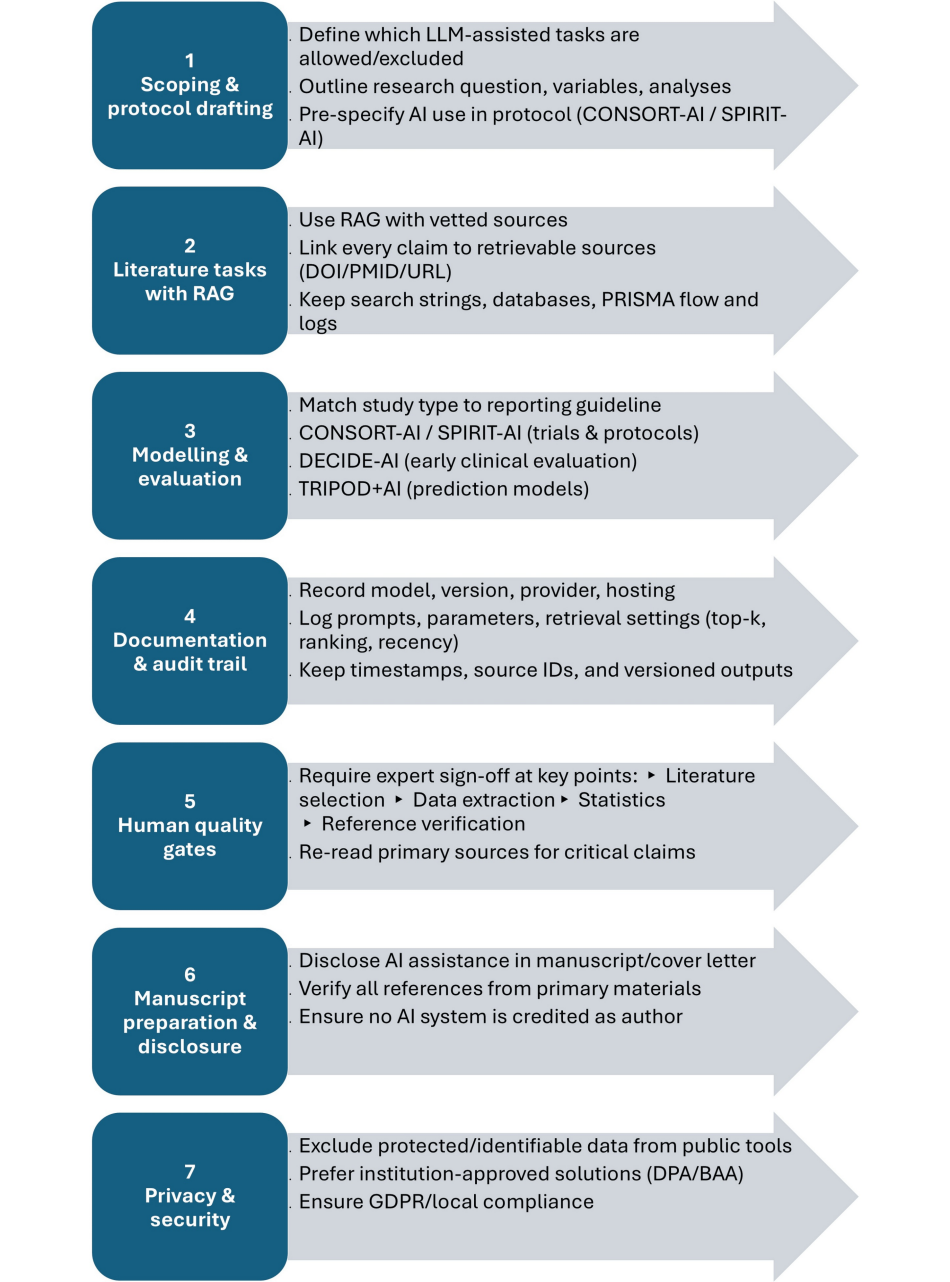
Seven-step workflow for LLM-assisted clinical trials and prediction models. Derived from reporting guidance for trials, protocols, and early evaluation (CONSORT-AI, SPIRIT-AI, and DECIDE-AI) [7-9] and from RAG literature (MIRAGE) [18,19]. AI: artificial intelligence, BAA: business associate agreement, DOI: Digital Object Identifier, DPA: data processing agreement, GDPR: General Data Protection Regulation, ID: identifier, LLM: large language model, PMID: PubMed Identifier, PRISMA: Preferred Reporting Items for Systematic Reviews and Meta-Analyses, RAG: retrieval-augmented generation, top-k: number of highest-ranked retrieved items considered in a RAG pipeline, URL: Uniform Resource Locator; SPIRIT-AI: Standard Protocol Items: Recommendations for Interventional Trials - Artificial Intelligence, TRIPOD+AI: Transparent Reporting of a Multivariable Prediction Model for Individual Prognosis or Diagnosis - Artificial Intelligence; CONSORT-AI: Consolidated Standards of Reporting Trials - Artificial Intelligence, DECIDE-AI: Developmental and Exploratory Clinical Investigations of Decision - Support Systems Driven by Artificial Intelligence Image Credits: Authors

A practical workflow for the process is illustrated in Figure 1.

Step 1 - Scoping and Protocol Drafting

Outline the research question, variables, and planned analyses; decide which LLM-assisted tasks are allowed and which are excluded; and pre-specify AI use in the protocol (CONSORT-AI/SPIRIT-AI for trials and protocols) [7,8]. Require manual verification for any claim drafted with LLM support.

Step 2 - Literature Tasks With RAG

Implement retrieval-augmented workflows so every claim links to retrievable primary sources (DOI (Digital Object Identifier)/PMID (PubMed Identifier)/URL (Uniform Resource Locator)). Maintain full search strings, databases, dates/limits, de-duplication criteria, and a PRISMA flow with screening logs (see Table 3) [11].

**Table 3:** Literature and retrieval checklist. These checklists are intended to be completed and filed with the study’s protocol and submission package. They support transparency and do not replace formal reporting guidelines (CONSORT‑AI, SPIRIT‑AI, DECIDE‑AI, and TRIPOD+AI). DOI: Digital Object Identifier, ID: identifier, PMID: PubMed Identifier, PRISMA: Preferred Reporting Items for Systematic Reviews and Meta-Analyses, RAG: retrieval-augmented generation, top-k: number of highest-ranked retrieved items considered in a RAG pipeline, URL: Uniform Resource Locator; SPIRIT-AI: Standard Protocol Items: Recommendations for Interventional Trials - Artificial Intelligence, TRIPOD+AI: Transparent Reporting of a Multivariable Prediction Model for Individual Prognosis or Diagnosis - Artificial Intelligence; CONSORT-AI: Consolidated Standards of Reporting Trials - Artificial Intelligence, DECIDE-AI: Developmental and Exploratory Clinical Investigations of Decision - Support Systems Driven by Artificial Intelligence

Checklist item	What to document (audit trail)	Pass criteria (Y/N/NA)
Approved corpora	List of vetted sources (databases, repositories)	Approved sources list/allow-list used
Search strategies	Full query strings, databases, dates, limits	Strategies saved/exported
Retrieval settings	Retrieval top-k, ranking, filters, recency bounds	Parameters recorded
De-duplication and quality	De-duplication method and source quality screening criteria	Methods recorded
Citation integrity	Every claim linked to retrievable primary sources (DOI/PMID/URL)	Links verified
Prompt and context logs	Prompts, retrieved contexts, outputs, timestamps, source IDs	Logs retained
Version control	Output versions with change history	Version control in place
Dual verification	Two-person check for critical claims/quantitative results	Sign-off recorded
PRISMA tracking (if review)	Screening log, inclusion/exclusion reasons, PRISMA flow	Complete
De-identification check	Confirm no protected/identifiable data in prompts/contexts	Confirmed
Error log	Known failure modes, hallucinations, corrective actions	Log maintained
Reproducible environment	Random seeds/hyperparameters (if modelling), tool versions	Environment captured

Step 3 - Modelling and Evaluation

Match the study type to the appropriate guidance: CONSORT-AI/SPIRIT-AI for AI-enabled interventions; DECIDE-AI for early, live clinical evaluation of decision support; TRIPOD+AI for clinical prediction model development/validation (see Table 4) [7-10]. Record performance metrics, calibration/uncertainty, and pre-specified subgroup/fairness analyses [13-16].

**Table 4:** Reporting and reproducibility checklist. These checklists are intended to be completed and filed with the study’s protocol and submission package. They support transparency and do not replace formal reporting guidelines (CONSORT‑AI, SPIRIT‑AI, DECIDE‑AI, TRIPOD+AI) CI: confidence interval, COI: conflict of interest, GDPR: General Data Protection Regulation, OSF: Open Science Framework, PI: prediction interval, PRISMA 2020: Preferred Reporting Items for Systematic Reviews and Meta-Analyses 2020, PROSPERO: International Prospective Register of Systematic Reviews, SPIRIT-AI: Standard Protocol Items: Recommendations for Interventional Trials - Artificial Intelligence, TRIPOD+AI: Transparent Reporting of a Multivariable Prediction Model for Individual Prognosis or Diagnosis - Artificial Intelligence; CONSORT-AI: Consolidated Standards of Reporting Trials - Artificial Intelligence, DECIDE-AI: Developmental and Exploratory Clinical Investigations of Decision - Support Systems Driven by Artificial Intelligence

Requirement	Applies to	Evidence to retain (audit trail)
Map to appropriate guideline	All studies	Rationale for selecting CONSORT-AI, SPIRIT-AI, DECIDE-AI, TRIPOD+AI, PRISMA 2020
Study registration/protocol	Interventional AI; systematic reviews	Trial/PROSPERO/OSF numbers; protocol version
Population and setting clarity	All studies	Clear eligibility, setting, timeframe
Outcomes and metrics pre-specification	All studies	Primary/secondary outcomes; performance metrics
Model description	Prediction models	Model family, features, training data summary
Validation strategy	Prediction models	Internal/external validation details
Calibration and uncertainty	Prediction models	Calibration plots; CI/PI reporting
Human factors and safety	Early clinical evaluation	Usability tasks, workflow fit, risk controls
Randomization and blinding	Interventional AI	Randomization, blinding, allocation concealment
Fairness/subgroup analyses	All with human data	Pre-specified subgroups; disparity metrics
Harms and error analysis	All studies	Adverse events; error taxonomies
Data governance and privacy	All with data	GDPR compliance statement; data sharing limits
Transparency of AI use	All manuscripts	Location of AI use disclosure in paper
Reproducibility	All	Code/data availability (when applicable); retrieval logs
Limitations	All	Limitations and generalizability

Step 4 - Documentation and Audit Trail

Record model/provider, version/date, hosting, and data residency; prompts, parameters, retrieval settings (top-k, ranking, and recency), and timestamps/source IDs; and keep versioned outputs. This enables reproducibility and post-hoc auditing [18].

Step 5 - Human Quality Gates

Require expert sign-off at key checkpoints: literature selection, data extraction, statistics, and reference verification. For high-impact claims (e.g., primary outcomes), mandate targeted re-reads of the primary sources before submission [10,11].

Step 6 - Manuscript Preparation and Disclosure

Disclose how and where AI was used (methods/acknowledgments/cover letter), verify all references against primary materials, and ensure no AI system is credited as an author, consistent with ICMJE/WAME/COPE [4-6].

Step 7 - Privacy and Security

Keep protected/identifiable information off public tools; prefer institution-approved solutions under the right agreements (e.g., data processing agreement (DPA)/business associate agreement (BAA); document the legal basis (e.g., GDPR) and any required approvals; and align with local institutional policy [1,20].

## Discussion

This technical report proposes a pragmatic workflow to integrate LLMs into clinical trials and prediction models while preserving scientific rigor. In practice, many failure modes trace back to weak grounding and insufficient documentation. To our knowledge, this is the first workflow that translates multiple international guidelines (CONSORT-AI, SPIRIT-AI, DECIDE-AI, TRIPOD+AI, and PRISMA 2020) into a unified, auditable process for daily clinical trials and prediction models (see Table 1 for a comparative overview). By pairing generation with retrieval from rights-cleared, authoritative sources (RAG) and inserting human “quality gates” at predefined checkpoints (Table 2 and Table 3), the workflow aims to mitigate hallucinations and fabricated citations and to ensure that claims are anchored to retrievable primary materials [12,14].

Several risks require explicit attention. Upstream, governance demands that teams define the intended use of LLM assistance and pre-specify subgroup analyses and performance metrics aligned with study design. Downstream, prospective evaluation and targeted re-reads of critical sources reduce the chance that biased or unstable signals survive to publication [10,11]. Beyond measurement error, unintended consequences of automation can arise when oversight is weak; explicit roles, responsibilities, and documented sign-off help counter over-reliance on automated outputs [13,14]. A further concern is the spread of so-called black box deep research approaches, where neither the provenance of outputs nor the internal reasoning of models is transparent. Such practices threaten reproducibility and accountability, underscoring the importance of audit trails, disclosure statements, and alignment with reporting standards.

The workflow is intentionally mapped to existing editorial and reporting expectations. ICMJE, WAME, and COPE converge on transparency about AI assistance and on human accountability; our disclosure pattern and non-authorship stance operationalize those norms inside the manuscript record and cover letter [4-6]. Privacy safeguards are likewise embedded: protected or identifiable information is kept off public tools, institution-approved solutions are preferred under appropriate agreements such as the DPA/BAA, and the legal basis (GDPR) is documented in the audit trail and protocol [1,20].

This work should be viewed in continuity with earlier reporting frameworks: whereas CONSORT-AI, SPIRIT-AI, TRIPOD+AI, PRISMA 2020, and DECIDE-AI each target specific study types, our contribution lies in extending them into a reproducible, auditable workflow for day-to-day practice. Rather than proposing a new standard, this manuscript consolidates and operationalizes dispersed recommendations, turning them into actionable steps. In that sense, it is not a protocol but a technical report that builds on existing guidance and makes it practically usable.

Strengths include alignment with widely adopted guidance across trials, protocols, early clinical evaluation, prediction models, and systematic reviews; reusable checklists (Table 2 and Table 3) that translate policy into concrete actions; and an auditable record of prompts, versions, and retrieval contexts that supports reproducibility. Limitations are that we did not benchmark performance empirically across tasks or models, and effectiveness depends on institutional adoption (access control, approved corpora, and staff time) and on the coverage/quality of the source repositories used for retrieval. Practical applicability would be further strengthened by case studies; future work should, therefore, pilot the workflow in real-world projects, such as systematic reviews, early-phase clinical trials, and clinical prediction model development, to quantify error reduction, time savings, and user acceptance [12,18].

## Conclusions

The use of LLMs in clinical trials and prediction models can be very useful, but only when supported by clear rules and proper documentation. In this report, we suggest a seven-step workflow that follows international standards and current editorial policies. It relies on checking the original sources, keeping a record of what was done, review by experienced researchers, and strict rules for handling data. By doing this, common problems such as biased outputs or made-up references can be spotted early, and the risk of treating machine text as final conclusions is reduced. The purpose is not to replace human judgment. Instead, the process is meant to save time while keeping results reproducible and trustworthy. Because the steps are modular, groups can adjust the workflow to suit their own projects.

## References

[1] (2025). World Health Organization. Ethics and governance of artificial intelligence for health: guidance on large multi-modal models. https://www.who.int/publications/i/item/9789240084759.

[2] (2025). UNESCO recommendation on the ethics of artificial intelligence. https://unesdoc.unesco.org/ark:/48223/pf0000381137.

[3] (2025). AI in health huge potential, huge risks. Paris: OECD.

[4] (2025). Recommendations for the conduct, reporting, editing, and publication of scholarly work in medical journals. https://www.icmje.org/recommendations.

[5] Zielinski C, Winker MA, Aggarwal R (2024). Chatbots, generative AI, and scholarly manuscripts: WAME recommendations on chatbots and generative artificial intelligence in relation to scholarly publications. Curr Med Res Opin.

[6] Committee on Publication Ethics (COPE (2025). Authorship and AI tools. 13 Feb.

[7] Liu X, Rivera SC, Moher D, Calvert MJ, Denniston AK (2020). Reporting guidelines for clinical trial reports for interventions involving artificial intelligence: the CONSORT-AI Extension. BMJ.

[8] Cruz Rivera S, Liu X, Chan AW, Denniston AK, Calvert MJ (2020). Guidelines for clinical trial protocols for interventions involving artificial intelligence: the SPIRIT-AI extension. Nat Med.

[9] Vasey B, Nagendran M, Campbell B (2022). Reporting guideline for the early-stage clinical evaluation of decision support systems driven by artificial intelligence: DECIDE-AI. Nat Med.

[10] Collins GS, Moons KG, Dhiman P (2024). TRIPOD+AI statement: updated guidance for reporting clinical prediction models that use regression or machine learning methods. BMJ.

[11] Page MJ, McKenzie JE, Bossuyt PM (2021). The PRISMA 2020 statement: an updated guideline for reporting systematic reviews. BMJ.

[12] Gallifant J, Afshar M, Ameen S (2025). The TRIPOD-LLM reporting guideline for studies using large language models. Nat Med.

[13] Obermeyer Z, Powers B, Vogeli C, Mullainathan S (2019). Dissecting racial bias in an algorithm used to manage the health of populations. Science.

[14] Cabitza F, Rasoini R, Gensini GF (2017). Unintended consequences of machine learning in medicine. J Am Med Assoc.

[15] (2025). Nature Editorial. Tools such as ChatGPT threaten transparent science; here are our ground rules for their use. Nature.

[16] Thorp HH (2023). ChatGPT is fun, but not an author. Science.

[17] Flanagin A, Bibbins-Domingo K, Berkwits M, Christiansen SL (2023). Nonhuman "authors" and implications for the integrity of scientific publication and medical knowledge. J Am Med Assoc.

[18] Lewis P, Perez E, Piktus A (2020). Retrieval-augmented generation for knowledge-intensive NLP tasks. arXiv.

[19] Xiong G, Jin Q, Lu Z, Zhang A (2024). Benchmarking Retrieval-Augmented Generation for Medicine. Findings of the Association for Computational Linguistics: ACL 2024.

[20] (2025). Regulation (EU) 2016/679 of the European Parliament and of the Council of 27 April 2016 on the protection of natural persons with regard to the processing of personal data and on the free movement of such data, and repealing Directive 95/46/EC (General Data Protection Regulation). Official Journal of the European Union.

